# Advances in the structure and function of the nucleolar protein fibrillarin

**DOI:** 10.3389/fcell.2024.1494631

**Published:** 2024-11-13

**Authors:** Xue Zhang, Wenxin Li, Shulan Sun, Yefu Liu

**Affiliations:** ^1^ Central Laboratory, Cancer Hospital of Dalian University of Technology, Liaoning Cancer Hospital & Institute, Shenyang, China; ^2^ Department of Hepatobiliary and pancreatic, Cancer Hospital of Dalian University of Technology, Liaoning Cancer Hospital & Institute, Shenyang, China

**Keywords:** fibrillarin, GAR structural domain, phase separation, cancer, nucleolar protein

## Abstract

Fibrillarin (FBL) is a highly conserved and well-researched nucleolar protein found in eukaryotes. Its presence was first identified in 1985 through protein immunoblotting analyses using antisera from patients with autoimmune scleroderma. Through immunoelectron microscopy, FBL was shown to be localized in the dense fibrillar component of the nucleolus, leading to the term “fibrillarin”. The FBL protein is composed of 321 amino acids and contains two significant functional domains: the GAR domain and the methyltransferase domain. It is expressed in the nucleolus of eukaryotes. This makes FBL one of the most studied nucleolar proteins. While methylation is not essential for cell survival, the FBL gene is crucial for eukaryotic cells, underscoring the importance of investigating additional functions that do not rely on FBL methylation. This review will primarily examine the protein structural domains of FBL and its classic methyltransferase activity. Additionally, our review will examine the importance of the eukaryote-specific GAR structural domain of FBL in regulating intracellular phase separation. Furthermore, this paper analyzes recent developments in the utilization of FBL in the study of pathogen infections and cancer research over the past decade.

## 1 Introduction

In 1985, a single protein band with a molecular weight of 34 kDa and a pI of 8.5 was identified through immunoblot analysis of nucleolar proteins using scleroderma antibodies. Subsequent electron microscopic immunocytochemistry revealed that the protein recognized by the scleroderma antiserum was specifically localized within the fibrillar region of the nucleolus, encompassing both the dense fibrillar and fibrillar center regions. As a result, this protein has been designated “fibrillarin” (Ochs et al., 1985). RNA blot analysis revealed that the mRNA in question has a length of approximately 1,300 nucleotides. Furthermore, protein sequence alignment revealed that 67% of the amino acids in human fibrillarin are conserved in yeast fibrillarin, whereas 81% are conserved in *Xenopus* fibrillarin (Aris and Blobel, 1991).

FBL has a highly conserved methyltransferase domain in both ancient and eukaryotic organisms, which lays the functional foundation for FBL. The ribosomal RNA (rRNA) methyltransferase FBL serves as a key regulator in various early stages of ribosome biogenesis (Tollervey et al., 1993; Yoshikawa et al., 2011). It can regulate rRNA cleavage and modulate NORs throughout the cell cycle (Schimmang et al., 1989; Snaar et al., 2000). FBL has methylation activity, which is responsible for the 2′-O-methylation (2′-O-Me) of ribosomal RNAs (rRNAs) and the methylation of histone H2A at glutamine 104 (Omer et al., 2002; Tessarz et al., 2014). The structural domain of the N-terminus is currently thought to have helped FBL adapt to the higher complexity of eukaryotes, but the principles and mechanisms by which this domain works remain to be experimentally demonstrated.

FBL is a protein highly expressed in pluripotent embryonic stem (ES) cells. Knockout of this gene led to significant delays in rRNA processing, growth inhibition, and apoptosis in mouse ES cells (Watanabe-Susaki et al., 2014). The knockout of FBL results in reduced nuclear size and lifespan extension (Tiku et al., 2017). The NCL-1/TRIM2/Brat tumor suppressor extends lifespan and limits nucleolar size in the major *C. elegans* longevity pathways as part of a convergent mechanism centered on the nucleolus. NCL-1 is critical for regulating nucleolus size and inhibiting FIB-1 expression, thereby affecting lifespan via multiple pathways. FIB-1 RNAi knockdown reduced nucleolar size and extended lifespan in wild-type worms. How cytosolic NCL-1 affects nucleolar function remains unclear, although evidence suggests that it regulates FIB-1 in part through its 3′ UTR (Yi et al., 2015). Furthermore, increased FBL expression is observed in mature oocytes with age-associated dysregulation of protein metabolism. Internally, elevated FBL expression promotes the proliferation and resistance of MCF-7 breast cancer cells to chemotherapy (Marcel et al., 2013). In addition, viral infections due to immune system dysregulation and tumors may present distinct disease spectra, but there are subtle and far-reaching associations between pathophysiology, genetic background, environmental factors, and therapeutic strategies (Masetti et al., 2021). In systemic scleroderma (SSc), it is not uncommon to find malignant tumors in patients with this disease. Although it is an infrequent nucleolar autoantibody, antifibrillarin is a marker for severe SSc (Arnett et al., 1996). FBL, as a nucleolar protein, is also a potential target for viral infection. This review starts from the structural domains of FBL and summarizes recent research on the functions of different structural domains of FBL.

## 2 Structure and function of FBL protein

**FIGURE 1:**
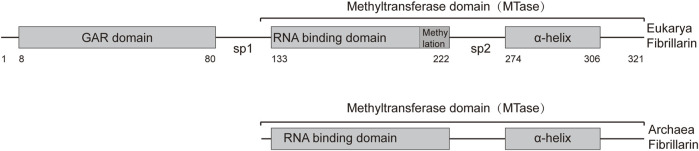
Representation of the primary structures of Archaea and Eukarya fibrillarin. The fibrillarin sequence is divided into three regions. The GAR domain is a sequence rich in glycine and arginine (8–80 aa). The methyltransferase domain contains the enzymatic activity as well as a conserved RNA binding sequence. This domain can be subdivided into an RNA-binding domain (133–222 aa) and the α-helix region (274–306 aa). Sp1 and Sp2 represent the first and second spacers, respectively. The average amino acid position of each domain is given below the bar.

The human fibrillarin molecule, consisting of 321 amino acids, comprises two primary structural domains: the glycine and arginine rich (GAR) domain (8–80 aa) and the methyltransferase domain (133–306 aa). The methyltransferase domain includes a sequence with presumed RNA-binding ability (133–222 aa) and an α-helix (274–306 aa) (Figure 1) (Guillen-Chable et al., 2020). FBLs exhibit conserved amino acid sequences and secondary structures, with methyltransferase domains highly conserved across archaea and eukaryotes.

**FIGURE 2:**
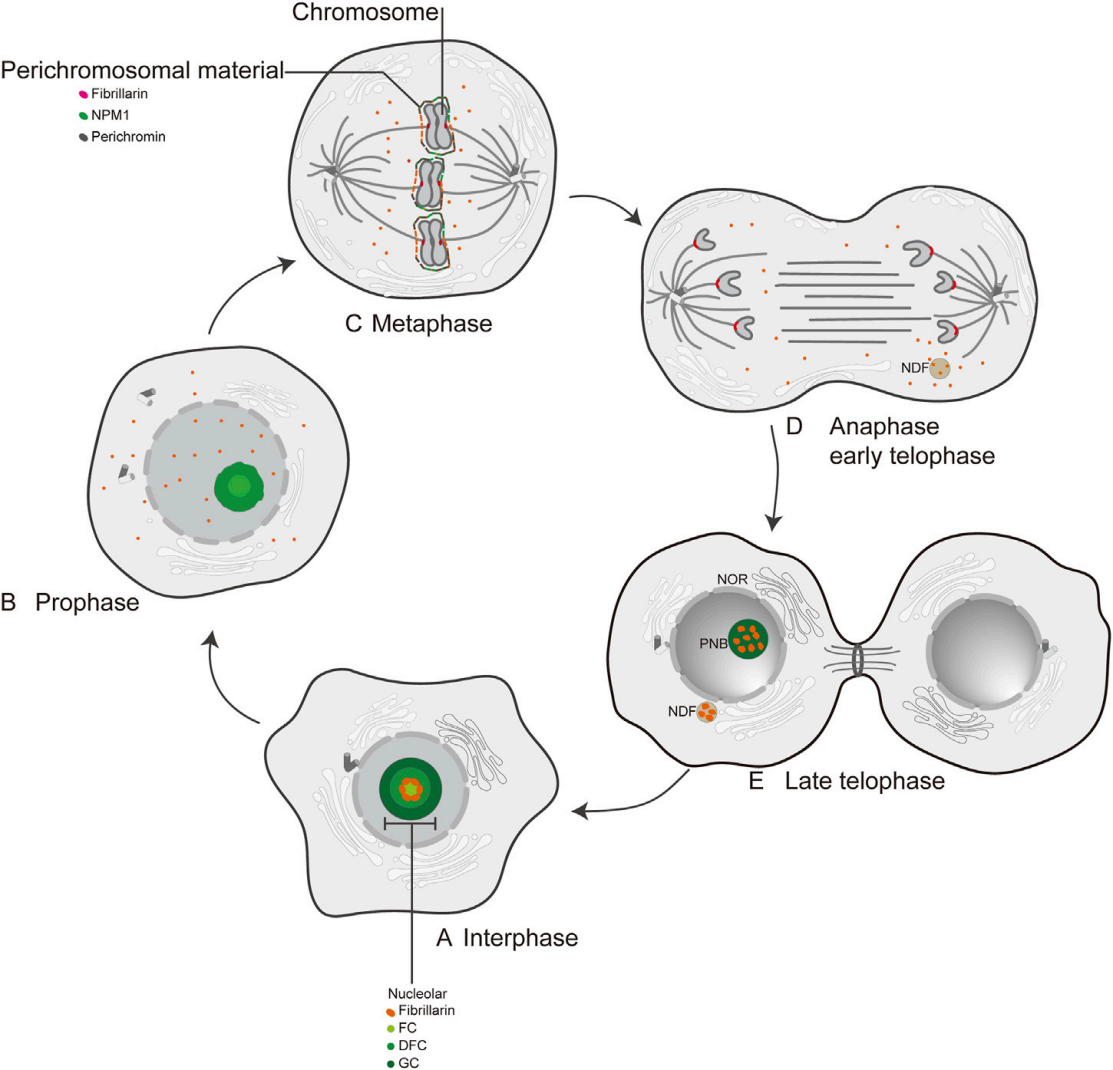
The localization of FBL at interphase and mitosis in human cell. **(A)** Interphase; **(B)** Prophase; **(C)** Metaphase; **(D)** Anaphase early telophase; **(E)** Late telophase; FC: fibrillar center; DFC: dense fibrillar component; GC: granular component; NPM1: nuclear phosphoprotein; Perichromin: nuclear matrix protein; Perichromosomal material: perichromosomal material; NDF: nucleolus-derived foci; NOR: nucleolar organization region; PNB: prenucleosomal body.

In human cells, FBL is predominantly localized in nucleolar fractions (Jansen et al., 1991; Pederson, 2011). Electron microscopic studies have identified three distinct regions within the nucleolus: the fibrillar center (FC), the dense fibrillar component (DFC), and the granular component (GC) (Pederson, 2011) (Figure 2A). Previous studies have reported the localization of FBL in pig PK cells (Mukharyamova et al., 1999) and human cervical cancer cells (HeLa) (Mukharyamova et al., 1999; Amin et al., 2007). During the early stages of the G1 phase and progression through G1 into the S phase, FBL predominantly localizes to the nucleolus at the FC/DFC interphase (Kim and Kwon, 2021), where it serves as the site for rRNA transcription (Tollervey et al., 1991; Yao et al., 2019) (Figure 2A). The human nucleolus consists of several dozen FC/DFC units, each containing 2–3 transcriptionally active rDNAs at the FC/DFC interphase. Pre-rRNA processing factors, such as FBL, form 18–24 clusters that further assemble into the DFC surrounding the FC (Yao et al., 2019) (Figure 2B). During mitosis, FBL colocalizes with various proteins, such as chromophilic proteins, chromophilic nucleolar proteins, and the nuclear protein NPM1, as well as RNA processing factors, to form the perichromosome layer (Hernandez-Verdun and Gautier, 1994; Kosykh and Chentsov, 2002; Pederson, 2011), which may contribute to chromosome structure (Van Hooser et al., 2005) (Figure 2C). During anaphase of mitosis, the nucleolus undergoes reassembly, and a portion of the FBL protein colocalizes with nucleolus-derived foci (NDFs) (Baran et al., 1995; Dundr et al., 2000) (Figure 2D) (Tripathi and Parnaik, 2008). Prenucleolar bodies (PNBs) fuse with the primary nucleoli (the latter being formed around the transcribed rDNA clusters), and this step occurs around the nucleolar organizer region (NOR) (Figure 2E). Consequently, FBL is recognized as an early indicator of newly formed nucleoli. Notably, the subcellular localization of FBL varies across different species, such as in *Giardia lamblia,* where FBL is dispersed on the nuclear surface (Narcisi et al., 1998). Furthermore, FBL has been identified in Cajal bodies (CBs) adjacent to the nucleolus (Snaar et al., 2000; Morris, 2008). CBs are commonly referred to as “nucleolus appendages” and are primarily responsible for snRNA processing within ribonucleoprotein particles (RNPs) (Sawyer et al., 2016).

Through examination of the subcellular localization patterns of full-length FBL and truncated FBL mutants fused with GFP in human U-2OS cells, Snaar et al. identified the second spacer domain (sp2) and carboxy terminal α-helix domain as key elements responsible for directing FBL to nucleolar transcription centers and CBs (Snaar et al., 2000), respectively. Furthermore, recent research by Shubina et al. suggested that the GAR domain of FBL integrates nuclear localization signals and nucleolar localization signals (NoLSs). Methylation of arginine residues within the GAR domain is essential for facilitating nuclear import, yet it concurrently diminishes the effectiveness of nucleolar retention through the NoLS (Shubina et al., 2020).

FBL functions as an S-adenosyl-L-methionine-dependent methyltransferase capable of methylating both RNA and proteins. Its primary roles include site-specific methylation of ribose moieties in 18S and 28S rRNAs (Incarnato et al., 2017), pre-rRNA transcript processing, methylation of histone H2A, and protein methyltransferase activity (Loza-Muller et al., 2015; Iyer-Bierhoff et al., 2018). Methyltransferases ensure the execution of multiple cellular functions (Cheng and Roberts, 2001), including altering the local spatial structure of rRNA molecules to optimize the protein translation efficiency of ribosomes (Zust et al., 2011; Ringeard et al., 2019). However, the absence of rRNA 2-O-Me modification in organisms does not directly lead to direct death (Monaco et al., 2018).

NOP1 is considered a homologous counterpart of FBL in yeast (Schimmang et al., 1989; Henriquez et al., 1990; Schimmang et al., 1989 demonstrated that affinity-purified antibodies against yeast NOP1 efficiently coimmunoprecipitate a minimum of seven small nuclear RNAs implicated in rRNA maturation (Schimmang et al., 1989). Haploid cells with a disrupted NOP1 gene copy number exhibit inviability, underscoring the essential role of NOP1 in cell growth. Previous investigations in yeast have highlighted the importance of NOP1 in pre-RNA processing (Tollervey et al., 1991). Pulse labeling of proteins demonstrated that strains depleted of NOP1 exhibited significant impairment of cytoplasmic ribosome production and a decrease in rRNA levels. Despite NOP1 depletion, the accumulation of seven tested snoRNAs, including U3, was not prevented; however, the levels of two species, U14 and snR190, decreased. The snoRNAs synthesized in the absence of NOP1 maintain trimethylguanosine (TMG) cap structures. Subnuclear fractionation and immunocytochemistry analyses indicate that these snoRNAs remain localized in the nucleolus (Tollervey et al., 1991). In the zebrafish model, the optic tectum and eye are severely affected by Fbl (human FBL homology) depletion, whereas the ventral regions of the brain are less impacted. These morphogenesis defects are associated with impaired neural differentiation and massive apoptosis. Polysome gradient experiments have shown that Fbl mutant larvae display defects in ribosome biogenesis and activity (Bouffard et al., 2018). These morphological abnormalities are linked to compromised neural differentiation and extensive apoptosis. Furthermore, polysome gradient experiments demonstrated that Fbl mutant larvae exhibit deficiencies in ribosome biogenesis and function. Flow cytometry analyses revealed distinct S-phase profiles between wild-type and mutant cells, indicating a disruption in S-phase progression (Bouffard et al., 2018). Interestingly, the observed phenotypes are not solely attributed to the methyltransferase activity of FBL, implying that FBL may have additional essential functions beyond methylation.

This review aims to examine the fundamental structure and functionality of FBL proteins and discuss recent developments in research in various contexts, such as viral infections, autoimmune diseases, reproductive development, cellular stress, and cancer.

## 3 Structural domains of FBL

### 3.1 Methyltransferase structural domains and 2′-O-Me modifications

The archaebacterial FBL possesses a single methyltransferase domain (MD), leading to its initial classification as a methyltransferase. In contrast, eukaryotic FBL is a crucial component of Box C/D small nucleolar ribonucleoprotein particles (snoRNPs) (Rodriguez-Corona et al., 2015) and plays a role in 2′-O-Me modification at more than 100 sites on pre-rRNAs (Erales et al., 2017; Guillen-Chable et al., 2020). The methyltransferase structural domain within FBL is highly conserved across species and conforms to the typical structure of AdoMet-dependent methyltransferases (Niewmierzycka and Clarke, 1999; Barneche et al., 2000; Wang et al., 2000; Cheng and Roberts, 2001).

**FIGURE 3:**
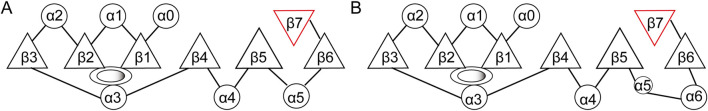
Structure of the methyltransferase catalytic domain. **(A)** Structure of the conserved methyltransferase catalytic domain of eukaryotic FBL. **(B)** Structure of the methyltransferase domain of fibrillarin-like protein from the archaeon *Aeropyrum pernix*. Circles, α-helices; triangles, β-strand; ovals: AdoMet binding sites.

The primary structural feature of methyltransferases is a central domain consisting of a seven-stranded β-sheet (β1-β7) surrounded by six α-helices. The β-sheet strands are organized in the sequence 6↓ 7↑ 5↓ 4↓ 1↓ 2↓ 3↓, with all strands except the seventh being oriented in parallel (Figure 3) (Shubina et al., 2016). The antiparallel orientation of the seventh strand, positioned between the fifth and sixth strands, is believed to be crucial for the functionality of this domain (Shubina et al., 2016).

**FIGURE 4:**
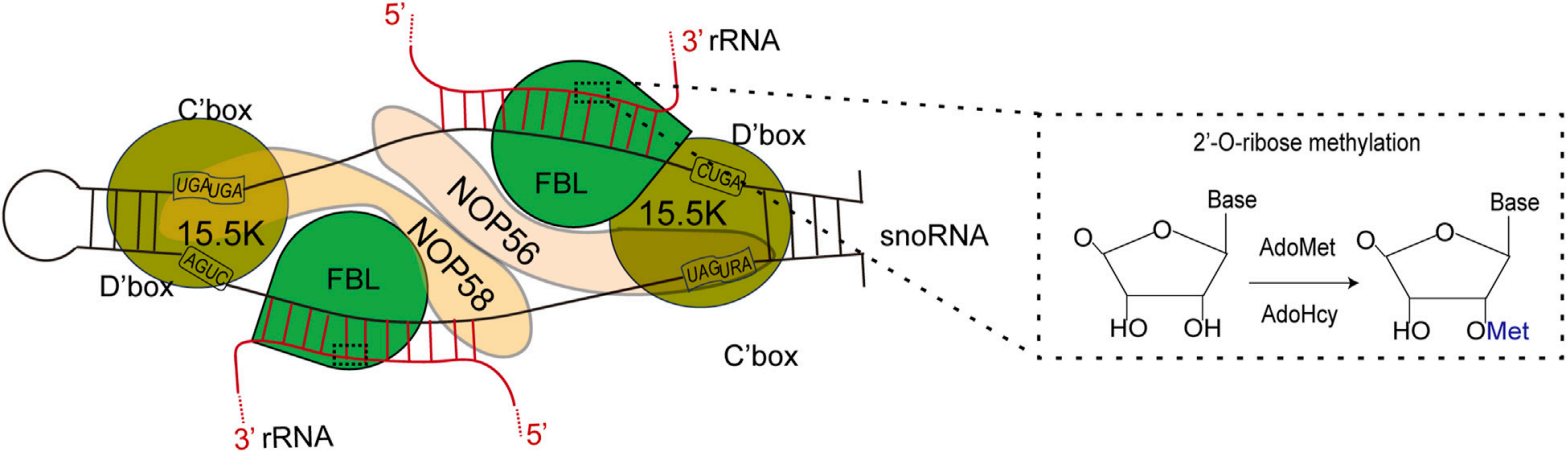
Left: FBL transforms S-adenosylmethionine (AdoMet) into S-adenosylhomoctene (AdoHcy) by catalyzing the methylation of its RNA substrate 2′-OH. Right: Schematic representation of the C/D box snoRNP complex, depicting the main features of the complex according to both biochemical data and structural studies using an archaeal complex. The C and D boxes are depicted with their consensus sequence. The substrate rRNA, red forms an RNA:RNA duplex with the snoRNA along 10–21 nucleotides (nt). The catalytic site of FBL faces the fifth nucleotide downstream of D′ boxes (yellow star). The 15.5K protein specifically binds a k-turn motif. rRNA: ribosomal RNA; snoRNA: small nucleolar ribonucleoprotein.

FBL primarily facilitates 2′-O-Me modifications on rRNAs with the assistance of BOX C/D small nucleolar RNAs (snoRNAs) (Rodriguez-Corona et al., 2015). FBL transfers the methyl group of SAM to the 2-hydroxyl group of the ribose target (Omer et al., 2002; Ye et al., 2009). 2′-O-Me is a prevalent form of RNA modification present in various RNA molecules, including rRNAs, tRNAs, snRNAs, and mRNAs (Ayadi et al., 2019). SnoRNPs are a critical component of the ribosome and spliceosome maturation process, representing one of the two major types of RNPs necessary for these maturation processes. A significant proportion of Box C/D snoRNPs are involved in the 2′-O-Me of ribosome and spliceosome RNA through an RNA-guided mechanism (Balakin et al., 1996; Kiss-Laszlo et al., 1996). Fibrillarin facilitates this methylation process by transferring the methyl group from a bound S-adenosyl-methionine (SAM) molecule to the 2′-hydroxyl group of the target RNA nucleotide (Peng et al., 2014). In 2013, Audrone Lapinaite conducted a study on the structure and function of the Box C/D sRNP complex of *Rhodococcus pyogenes* in solution via a combination of solution-state NMR and small-angle X-ray with neutron scattering techniques (Lapinaite et al., 2013). The process involves the coordination of Nop56/58 in directing both rRNA and FBL, followed by the targeting of the catalytic subunit to a specific site on the target rRNA to facilitate methylation (Figure 4) (Filipowicz and Pogacic, 2002; Monaco et al., 2018). Like prokaryotes, eukaryotes form the NOP56:NOP58 heterodimer (two long alpha helices), which spans the snoRNA that spans the snoRNA and binds specifically to the terminal C/D and internal C'/D′ RNA motifs. The rRNA complex helps lock FBL in its active position (Yang et al., 2020).

**FIGURE 5:**
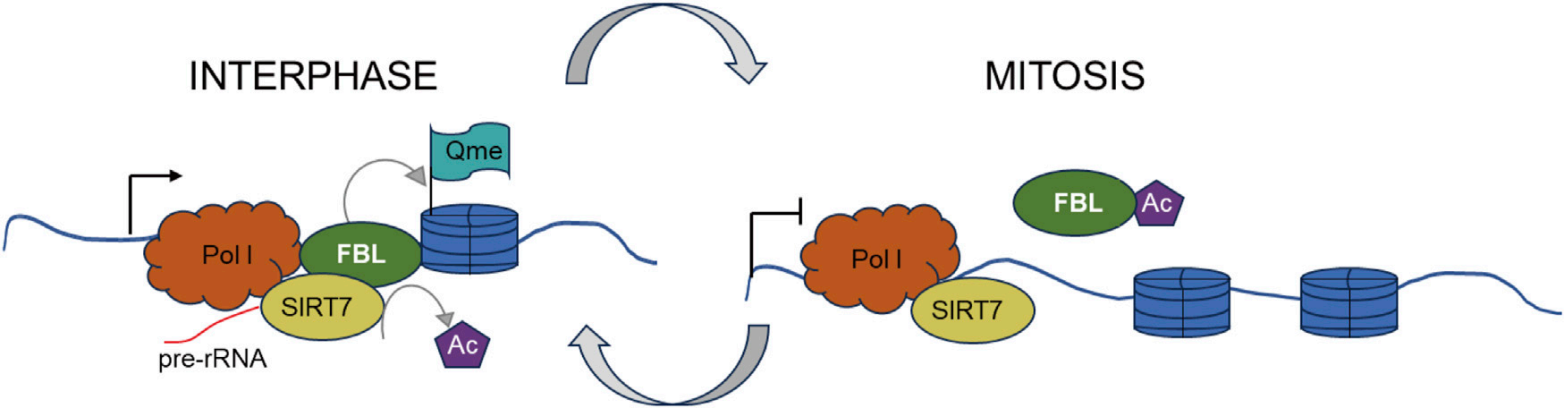
SIRT7-dependent deacetylation of fibrillarin controls histone H2A methylation and rRNA synthesis during thFe cell cycle. Left. At interphase, SIRT7-dependent deacetylation of FBL ensures a high level of H2AQ104me and rRNA synthesis during interphase. Right. At the onset of mitosis, cellular nucleus disassembly is accompanied by a high level of FBL acetylation, H2AQ104me loss, and is accompanied by inhibition of Pol I transcription.

Furthermore, in mammalian, yeast, and plant cells, FBL has been identified as a methyltransferase that targets histone H2A at glutamine (Q) 104, Q105, and Q106, respectively (Knight et al., 2014; Iyer-Bierhoff et al., 2018). This methylation of histone H2A glutamine has been shown to be positively associated with the transcription of rRNA by RNA polymerase I (Pol I) (Tessarz et al., 2014; Loza-Muller et al., 2015). During mitosis, the disassembly of the nucleolus is accompanied by increased acetylation of FBL, loss of H2AQ104 methylation, and inhibition of Pol I transcription. The restoration of H2AQ104me and transcriptional activity is observed in cells overexpressing an acetylation-deficient FBL mutant but not in those overexpressing an acetylation-mimicking mutant. Iyer-Bierhoff et al. illustrated this dynamic mechanism in a hypothetical diagram in their original article (Figure 5) (Tessarz et al., 2014; Loza-Muller et al., 2015).

### 3.2 Ribonuclease activity of FBL

Ribonucleases play crucial roles in various cellular processes, such as cytoplasmic and nuclear RNA degradation, RNA interference, antiviral defense, DNA synthesis, and RNA processing. In 2004, Vasquez Saez et al. identified a ribonucleoprotein complex, named nuclear Factor D (NF D), composed of 30 proteins in *Cruciferae*, including FBL, nucleolin, and snoRNAs U3 and U14. This complex has been found to interact with rDNA and cleave the P site located downstream of the A1, A2, A3, and B sequences in the 5′ ETS of pre-rRNA (Saez-Vasquez et al., 2004).

Tollervey et al. reported that conditional knockout of NOP1 in yeast resulted in a reduction in overall rRNA levels and an accumulation of unprocessed rRNA molecules (Tollervey et al., 1991). Recent research conducted by Guillen-Chable et al. revealed that FBL has additional ribonuclease activity, and researchers have shown that ribonuclease activity is localized to the glycine/arginine-rich (GAR) domain conserved in a small group of RNA-interacting proteins (Guillen-Chable et al., 2020). This study demonstrated that the ribonuclease activity of FBL was enhanced by phosphatidylinositol 5-phosphate (PI5P) and attenuated in the presence of phosphatidyl acid (PA) or upon binding to the guide RNA U3 (Guillen-Chable et al., 2020).

Numerous nucleolar proteins, including Nucleolin (Lapeyre et al., 1987), NSR1 (Lee et al., 1991), SSB1 (Jong et al., 1987), and GAR1 (Girard et al., 1992), possess GAR domains similar to those in FBL, enabling them to directly interact with rRNA and contribute to its processing.

### 3.3 GAR structural domains of FBL with phase separation

**FIGURE 6:**
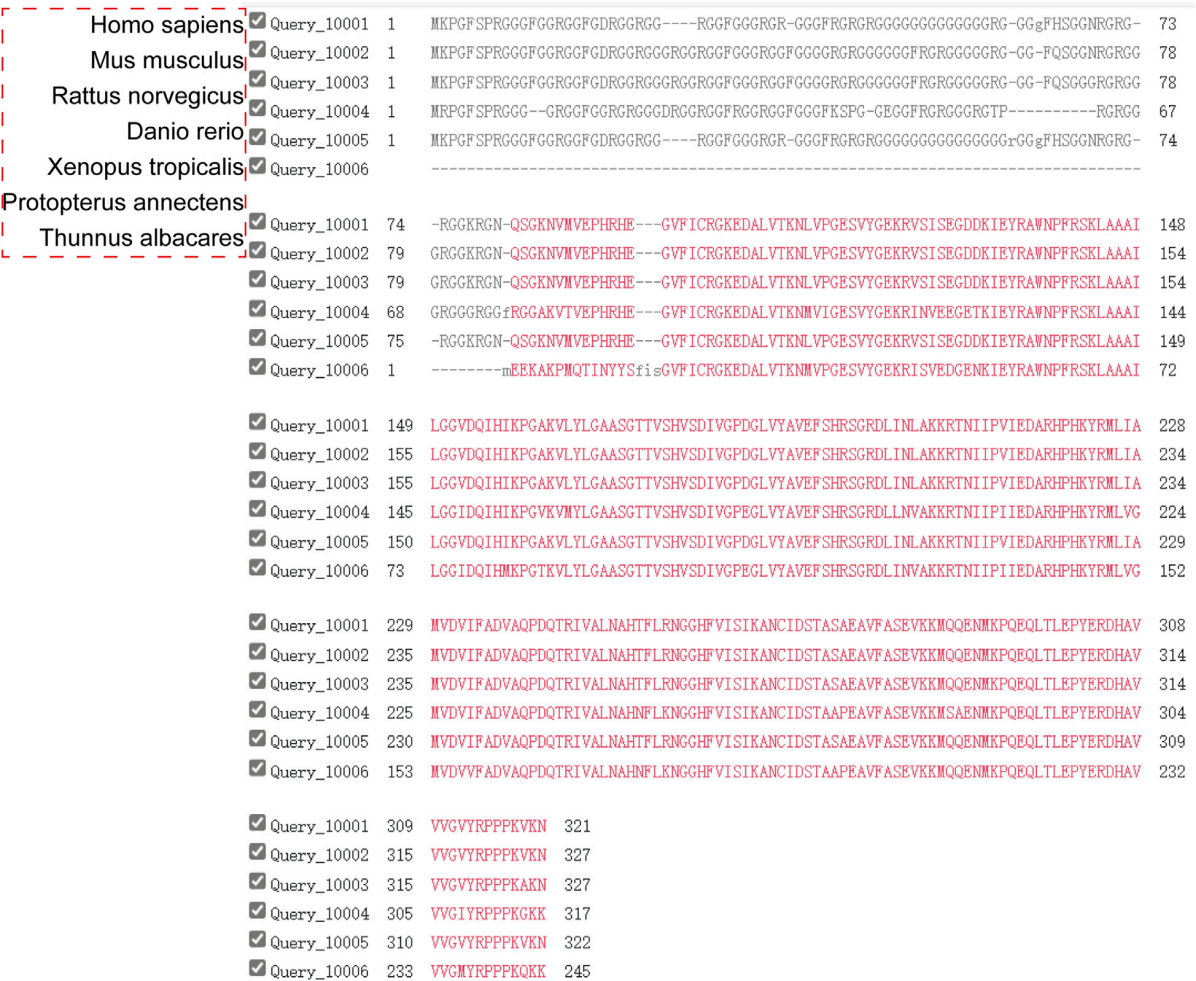
Multiple alignment of amino acid sequences of fibrillarins from different taxonomic groups. The RNA-binding and the α-helical domains of fibrillarins are characterized by a high degree of homology.

The N-terminal domain of fibrillarin is conserved in eukaryotes but not in archaea. This region, known as the GAR domain, consists of approximately 85 amino acids rich in glycine and arginine residues (David et al., 1997). The derived amino acid sequence of the N-terminal region of *Tetrahymena* fibrillarin shows little similarity with the generally highly conserved glycine/arginine-rich N-terminal domain of other eukaryotic FBLs. The N-terminal domains of *T. thermophila* and *G. lamblia* exhibit notable divergence from the N-terminal domains of fibrillarins found in other organisms, including animals and plants (Shubina et al., 2016). We further compared the fibrillarin proteins of *Homo sapiens、Mus musculus、Rattes norvegicus、Danio rerio、Xenopus tropicalis、Protopterus annectens* and *Thunnus albacares*. As expected, they all have a conserved C-terminal structural domain and a variable GAR structural domain (Figure 6).

Decades ago, motifs abundant in arginines and glycines were identified as playing functional roles and were designated as GAR domains and Arg-Gly-Gly (RGG) boxes. It has been established that proteins containing glycine and arginine-rich domains are integral to biological systems (Chandana and Venkatesh, 2016). These low complexity repetitive sequences give rise to intrinsically disordered regions (IDRs), providing proteins with increased conformational flexibility and adaptability, facilitating their ability to bind to diverse targets. Over 1,000 human proteins contain the RGG/RG motif, impacting various physiological processes including transcription, pre-mRNA splicing, DNA damage signaling, mRNA translation, and apoptosis regulation (Thandapani et al., 2013). The GAR structural domain found exclusively in eukaryotic FBLs is now recognized as a significant factor in determining the subcellular localization (Snaar et al., 2000) of FBLs and promoting phase separation. The intrinsically disordered region of the GAR structural domain interacts with a wide range of biomolecules (Pellizzoni et al., 2001) and is involved in processes such as metabolism of snoRNPs (Bracha et al., 2019) as well as viral infection (Klarnet et al., 1989; Snaar et al., 2000).

According to reports, FBL “diffuses” (a process that resembles diffusion) within the DFC of the nucleolus, where its GAR domains facilitate local self-association to create phase-separated clusters. These clusters serve to immobilize FBL-interacting pre-rRNA molecules, thereby promoting the directional movement of nascent pre-rRNA and facilitating both pre-rRNA processing and DFC formation (Yao et al., 2019). Membraneless organelles exhibit liquid-like behavior, a characteristic of liquid‒liquid phase separation (LLPS) (Falahati and Haji-Akbari, 2019; Strom and Brangwynne, 2019; Zhu et al., 2019). Membraneless organelles are rich in proteins, RNA molecules and lipids, which, especially proteins, are regulated to undergo various modifications, which in turn increase their tendency to interact with other internal components (Strom and Brangwynne, 2019; Riback et al., 2020). Wang Yichun et al. demonstrated the classification of lengthy GAR motifs and their potential relationships with protein/RNA interactions and liquid‒liquid phase separation via the GMF algorithm (Wang et al., 2023).

The GAR structural domain of FBL includes IDRs (Novak and Mitchell, 1988; Rodriguez-Corona et al., 2015), which have been the focus of numerous studies because of their potential role in phase separation. Research suggests that IDRs may be essential for phase separation, particularly when they are present at high concentrations *in vitro* (Burke et al., 2015; Monahan et al., 2017). Additionally, investigations have shown that longer IDRs within the GAR region of FBL can enhance self-association of FBL in cells, as demonstrated by increased polymerization of FBL mutants on native gels (Yao et al., 2019). The GAR structural domain of FBL features multiple R/G and phenylalanine/glycine (FG) residues that can interact with a diverse array of proteins and RNAs (Hofler et al., 2021). This biomolecular interaction likely serves as the underlying mechanism for the formation of LLPS (Boeynaems et al., 2018). Jiwon Lee et al. reported that the presence of phenylalanine residues within the IDRs of FBL is essential for the phase transition process, as evidenced by the failure of FBL with an F-to-S mutation in the IDRs to localize in the DFC regions of the nucleoli, similar to intact fibrillarin. Furthermore, the phase transition of the fibrillarin IDR domain is essential for its interaction with other RNA-binding proteins, including FUS, TAF15, DDX5, and DHX982 (Kato et al., 2012). Numerous studies have highlighted the importance of IDRs, which can play a crucial role in phase separation, particularly when they are present at high concentrations *in vitro* (Burke et al., 2015; Brady et al., 2017; Monahan et al., 2017).

The significance of phase separation in RNA-associated biological processes is evident (Kim and Kwon, 2021; Lin and Fang, 2021), as demonstrated by the presence of RNA-binding proteins with IDRs such as FBL (Shubina et al., 2020; Yoneda et al., 2021; Okuda et al., 2022), NPM1 (Hisaoka et al., 2014; Mitrea et al., 2018), Nucleolin (Lapeyre et al., 1987), NSR1 (Lee et al., 1991), SSB (Jong et al., 1987), and GAR1 (Girard et al., 1992), which interact with rRNAs to form complexes involved in the regulation of transcription (Shao et al., 2022). A previous study revealed that FBL cotranscriptionally binds to the 5′ end of nascent pre-rRNA transcripts (the so-called 5′ external transcribed spacer, or 5′ETS) and that its amino-terminal GAR domain containing IDRs promotes RNA phase separation, leading to DFC formation and initial RNA processing (Lafontaine, 2019).

The nucleolus contains a significant proportion of RNA-binding proteins (RBPs), with over half of its proteins being RBPs. In addition to facilitating RNA interactions, IDRs within these RBPs are subject to regulation through posttranslational modifications (PTMs) (Zhou et al., 2018; Modic et al., 2024). Current evidence suggests that the phosphorylation and methylations of IDRs and the interplay between various PTMs play crucial roles in regulating molecular interactions and facilitating phase separation (Luo et al., 2021).

### 3.4 GAR structural domains interact with viral proteins

Many viruses promote the infection process by interacting with the nucleolus and its components (Greco, 2009). Research indicates that viruses may specifically target the nucleolus and its components to increase viral transcription and translation and potentially manipulate the cell cycle to facilitate viral replication (Hiscox, 2002). FBL is a key target of certain plant and animal viruses, with viral-encoded proteins containing IDRs such as nucleocapsids, matrix proteins, Tat proteins, and even viral snoRNAs associated with FBL to induce unconventional functions in the nucleolar protein. Viruses play a crucial role in the transportation of viral particles within infected cells by interacting with the FBL protein, thereby influencing its subcellular localization to facilitate viral particle transport. In plants, the N-terminal domain of *Arabidopsis thaliana* Fib (human FBL homology) is segmented into the GAR domain and a spacer region, with various cellular and viral proteins showing a propensity to interact with the GAR structural domains (Decle-Carrasco et al., 2021). Rodriguez-Corona et al. (2017) find a novel ribonuclease activity within the AtFib2 GAR domain, but not in AtFib1. Recently, similar studies revealed that this novel activity is conserved in the GAR domain of *Homo sapiens* fibrillarin (Guillen-Chable et al., 2020).

Research has indicated that the nucleocapsids of various mammalian viruses, such as *white spot syndrome virus* (WSSV) (Xing and Shi, 2011), infectious *bronchitis virus* (IBV) (Chen et al., 2002), *mouse hepatitis virus* (MHV) (Wurm et al., 2001), porcine reproductive and *respiratory syndrome virus* (PRRS) (Wurm et al., 2001), and *severe acute respiratory syndrome coronavirus* (SARS-CoV) (Li et al., 2005; You et al., 2005), colocalize within the nucleus.

*Hendra virus* (HeV) and *Nipah virus* (NiV), both members of the genus Henipavirus within the family *Paramyxoviridae,* exhibit a unique ability among *paramyxoviruses* to cause severe infections in a wide range of animals and fatal diseases in both humans and animals (Croser and Marsh, 2013). A genome-wide siRNA screen conducted at biosafety level 4 identified 585 human proteins essential for *henipavirus* infection, many of which are associated with ribosome biosynthesis, nuclear export, nuclear import, and transcriptional regulation (Deffrasnes et al., 2016). FBL has a significant influence, particularly on the infection processes of measles, mumps, and respiratory syncytial viruses (Deffrasnes et al., 2016). Cells deficient in fibrillarin displayed substantial hindrance of *Henipavirus* RNA and protein synthesis, underscoring its essential involvement in the RNA replication stage of infection (Deffrasnes et al., 2016). The interactions between some of the viral proteins and FBL are listed in this paper (Table 1). This article details other interactions of plant viruses with FBL (Decle-Carrasco et al., 2021).

**TABLE 1:** List of FBL interactions with different viral proteins or RNAs (representative references in parentheses).

Genus	Virus	Protein/RNA	Reference (s)
*Potyvirus*	Potato virus A (PVA)	VPg	Rajamaki and Valkonen (2009)
*Potexvirus*	Bamboo mosaic virus (BaMV)	P20	Chang et al. (2016)
*Tenuvirus*	Rice stripe virus (RSV)	p2	Zheng et al. (2015)
*Necrovirus*	Beet black scorch virus (BBSV)	p7a	Wang et al. (2012)
*Hordeivirus*	Poa semilatent virus (PSLV)	TGB1	Semashko et al. (2012)
*Pomovirus*	Potato mop-top virus (PMTV)	TGB1	Lukhovitskaya et al. (2015)

The role of FBL in viral infection extends beyond binding viral proteins, as demonstrated by functional screening, which identified RNA 2′-O-Me FBL as a facilitator of viral infection. Li Panpan et al. reported an increase in 2′-O-Me on poly A^+^ RNA in virus-infected macrophages (Li et al., 2022). These findings have improved our understanding of the specific role of FBL in antiviral therapy and can potentially inform the development of novel antiviral therapeutics.

### 3.5 Asymmetric dimethylation of GAR structural domains

Arginine methylation is a crucial factor in multiple cellular processes, including RNA and protein interactions (Rajpurohit et al., 1994; Friesen et al., 2001; Mowen et al., 2001), signal transduction (Rajpurohit et al., 1994; Lin et al., 1996), transcriptional regulation (Wang et al., 2001), and subcellular localization (Shen et al., 1998; Nichols et al., 2000). The nucleolar protein is extensively methylated within its GAR structural domain, with this modification being linked to its nuclear localization (Guillen-Chable et al., 2020; Shubina et al., 2020).

The guanidinium group of arginine residues undergoes modification to yield ω-NG-mono-methyl-arginine (MMA), ω-NG, NG-asymmetric di-methyl-arginine (aDMA), or ω-NG, NG-symmetric di-methyl-arginine (sDMA) (Li et al., 2021). Studies have shown that RNA-binding proteins, particularly those containing arginine-rich RGG repeat sequences, predominantly exhibit MMA or aDMA modifications (Chen et al., 2011; Yagoub et al., 2015). Protein arginine methyltransferases (PRMTs) are categorized into three distinct types based on their production of final methylarginine products: type I PRMTs, such as PRMT1, 2, 3, 4, 6, and 8, facilitate the synthesis of MMA and aDMA; type II PRMTs, including PRMT5 and PRMT9, are responsible for the generation of MMA and sDMA; and type III PRMTs, specifically PRMT7, catalyze the production of MMA (Guccione and Richard, 2019). Following translation, arginine residues located within the GAR structural domain in FBL are predominantly subject to modification through asymmetric arginine demethylation (Najbauer et al., 1993; Ai et al., 1999). The human FBL protein has been found to contain 4.1 mol% NG, NG-dimethylarginine. Furthermore, a recombinant protein consisting of the N-terminal 182 amino acid residues of FBL fused with GST serves as a substrate for PRMT1 (Tang et al., 1998).

*In vitro* studies have shown that the GAR region within FBL proteins serves as a substrate for various PRMT (Zurita-Lopez et al., 2012) enzymes, as indicated in Table 2. The biological implications of arginine methylation within the GAR domain of FBL, particularly in relation to its cellular localization, remain uncertain. Overall, asymmetric arginine dimethylations play a role in signal transduction, nuclear localization, and interactions with nucleic acids (Zurita-Lopez et al., 2012). Additionally, the methylation of arginine residues in eukaryotic proteins has the potential to regulate their interactions with RNA (Zhao et al., 2008; Cho et al., 2012).

**TABLE 2:** Recognition by mammalian PRMTs of the protein methyl-accepting substrates utilized in this study (+, methylation observed; −, methylation not observed; representative references are in parentheses).

Name	PRMT1	PRMT2	PRMT3	PRMT4	PRMT5	PRMT6	PRMT7	PRMT8
GAR	^+^ Tang et al. (1998), Rho et al. (2001), Frankel et al. (2002), Lee et al. (2005), Miranda et al. (2005), Swiercz et al. (2005), Lakowski and Frankel (2009), Bonham et al. (2010), Cha et al. (2011)	^+^ Lakowski and Frankel (2009)	^+^ Tang et al. (1998), Frankel and Clarke (2000), Cheng et al. (2004), Singh et al. (2004), Miranda et al. (2005), Swiercz et al. (2005), Bonham et al. (2010), Castellano et al. (2010)	^−^ Frankel et al. (2002), Lee et al. (2005), Cha et al. (2011)	^+^ Branscombe et al. (2001), Rho et al. (2001), Pesiridis et al. (2009)	^+^ Frankel et al. (2002), Cheng et al. (2004), Miranda et al. (2005), Bonham et al. (2010), Cha et al. (2011)	^+^ Gary and Clarke (1998), ^−^ Miranda et al. (2004)	^+^ Lee et al. (2005), Sayegh et al. (2007), Bonham et al. (2010)

Arginine methylation of the GAR structural domain has been linked to the nuclear localization of FBL (Guillen-Chable et al., 2020; Shubina et al., 2020), but the nucleolar localization of FBL is contingent not only on the GAR domain (Snaar et al., 2000; Levitskii et al., 2004).

## 4 FBL and cellular homeostasis

The nucleolus serves as the primary site for ribosome synthesis and is a crucial organelle for regulating internal homeostasis (Lo et al., 2006; Raska et al., 2006; Cmarko et al., 2008; Hernandez-Verdun et al., 2010). FBL plays a significant role in modulating cellular stress responses and maintaining intracellular stability.

The nucleolus is involved in the transcription and processing of ribosomal RNA, as well as the assembly of ribosome subunits (Boulon et al., 2010). Disruption of ribosome biosynthesis at any stage may affect the occurrence of nucleolar stress (NS) (Boulon et al., 2010), which is characterized by the nucleoplasmic translocation of nucleolar proteins (Yang et al., 2016). During UV irradiation, the nucleolus is not completely disrupted, but nucleolar proteins (RNAP1, FBL) and nucleolar DNA are exported to the periphery of the nucleolus (for simplicity, this phase is called “displacement”), and when DNA repair is complete, the correct nucleolar structure is restored (for simplicity, this phase is called “repositioning”) (Daniel et al., 2018). Several structures and nucleolar proteins that are involved in the repair process of this nucleolar integrity mechanism have been studied. One such protein is FBL (Musawi et al., 2023). Research indicates that a subset of nucleolar proteins, such as FBL, participate in the formation of “nucleolar caps”, which are structures formed typically during DNA damage (Jones, 2008; Boulon et al., 2010; Castle et al., 2012; Mironova et al., 2014; Zhang et al., 2014; Rosinska and Filipek, 2018). Recent studies suggest that various nucleolar proteins, including FBL, may function as cellular “stress sensors” to modulate cellular homeostasis. In response to stress, FBL translocates from the nucleolus to the nucleoplasm, where it forms speckles that colocalize with coilin, a marker of CBs (He et al., 2018). When cells undergo stress, such as DNA damage, the nuclear phosphoprotein NPM1 is activated to translocate from the nucleolus to the nucleoplasm. This translocation serves to stabilize p53 levels and facilitate the activation of p53-dependent expression of genes involved in cellular adaptive responses (Raska et al., 2006). The translocation of FBL may be facilitated by NPM1, a nuclear phosphoprotein located in the granular region of the nucleolus, through a mechanism that remains unknown. Investigations into the role of FBL in maintaining cellular homeostasis are ongoing, and numerous unresolved mechanisms have been investigated.

## 5 FBL and its role in germ cells

The chromatoid body is a specialized organelle found in male reproductive cells, specifically in spermatocytes and spermatids (Yokota, 2008). It plays a crucial role in the process of sperm formation and differentiation by facilitating RNA metabolism and the storing of proteins (Head and Kresge, 1985; Chuma et al., 2006). In late spermatids, the caudal cytoplasmic mass contains flower-like structures known as chromatoid bodies. Upon detachment of the caudal cytoplasm from the free spermatozoa and the formation of the residual body, the chromatoid body is no longer present (Breucker et al., 1985). In spermatids, the chromatoid body forms a temporary and close association with clusters of nuclear pores, indicating potential interactions between the chromatoid body and the nucleus (Fawcett et al., 1970). Research findings suggest that in early spermatids, the chromatoid body was observed in conjunction with Golgi cisterns, suggesting potential involvement in the acrosome formation process. In late spermatids, the centriolar body was observed in close proximity to the axonema, indicating a potential role in facilitating the development of the spermatozoon tail (Peruquetti et al., 2008; Peruquetti et al., 2010). Previous studies have implicated the chromatoid body in the repositioning of nucleolin, suggesting its possible involvement in the nucleoplasmic transfer of ribosomal subunits (Sato et al., 2001; Beaudoin et al., 2009).

Through the analysis of FBL expression in the cytoplasmic bodies of testicular spermatogonial cells from *Triatoma sordida* and *Triatoma infestans*, it was determined that FBL is localized within the nucleus and certain cytoplasmic regions of germ cells during the spermatogenesis process in *Triatomines*. Furthermore, examination of FBL expression at various stages of mammalian spermatogenesis revealed a notable increase in expression levels during stages IV-VI of the seminiferous tubules. Immunofluorescence studies demonstrated colocalization of FBL with MIWI (Yuan and Zhao, 2017), a protein involved in RNA metabolism, and HSP70 (Eddy, 1999), a protein implicated in proteasomal folding. Subsequent immunoprecipitation experiments revealed that FBL exhibited binding affinity with MIWI, whereas no interaction with HSP70 was observed. These findings suggest that FBL may be a constituent of the chromatoid body and is involved in the regulation of spermatogenesis through its interactions with other chromatoid body components involved in RNA metabolism (de Pauli et al., 2017). Additionally, FBL has been implicated in oocyte development. Wang et al. demonstrated that FBL-GFP localization in the nucleolus differs between unsurrounded and surrounded nucleolus oocytes, with uneven and cloudy distributions in the former and even distributions with a few bright dots in the latter. Thus, nonsurrounded nucleolus (NSN) oocytes presented significant nascent RNA transcription activity compared with the transcriptionally quiescent surrounding nucleolus (SN) group (Wang and Na, 2021). Additionally, early development necessitates the presence of FBL (Bouffard et al., 2018), a protein that is highly expressed in pluripotent embryonic stem cells and plays a crucial role in regulating the differentiation potential of these cells (Watanabe-Susaki et al., 2014).

## 6 FBL, infection and autoimmunity

A novel function of the nucleolus in innate immunity has been recently discovered, wherein the downregulation of fibrillarin and nucleolar contraction enhances pathogen resistance across various taxa (Chen et al., 2020). This protective effect, which is mediated by decreased levels of FBL, appears to operate independently of the primary immune response pathway in *Caenorhabditis elegans* (Tiku et al., 2018). Pathogenic infections have been shown to decrease nucleolar size, ribosomal RNA, and fibrillarin levels. Bacterial infections reduce nucleolar size and fibrillarin levels in mammalian cells. Knockdown of fibrillarin prior to infection has been shown to increase intracellular bacterial clearance, reduce inflammation, and increase cell survival. These findings suggest a conserved role of fibrillarin in infection resistance (Tiku et al., 2018).

The clinical associations of anti-Th/To, PM-Scl, FBL/U3RNP and RNA polymerase I with scleroderma or scleroderma overlap syndrome are well established (Tall et al., 2017). Scleroderma overlap syndrome is well established, and commercial assays are being developed (Satoh et al., 2022). *In vivo* experiments have demonstrated that mercury alters FBL through direct binding or mercury-induced hydrolytic cleavage, leading to the recognition of modified (Kubicka-Muranyi et al., 1996; Pollard et al., 1997) FBL as a foreign protein by T cells and a subsequent immune response (Pollard et al., 2000). Neuroinflammation occurs in response to central nervous system (CNS) injury, infection, stimulation by toxins or autoimmunity. FBL mRNA and protein expression were detected in HT22 cells in the LPS-induced neuroinflammation model. Detailed mechanistic studies revealed that when c-Fos, AP-1 and SOS1 were inhibited, FBL expression decreased, whereas FBL expression increased when KRAS agonists were used. In addition, the transcript levels of inflammatory genes in the NF-kB pathway (including CD14, MYD88, TNF, TRADD and NFKB1) were increased after FBL overexpression (Zhang et al., 2024). Furthermore, FBL interacts with the nuclear protein SUNA1 to regulate salicylic acid levels and modulate pre-rRNA production, thereby increasing the translation efficiency of specific defense genes in Arabidopsis during infection with the pathogen *Pseudomonas syringae* (Pst DC3000) (Kong et al., 2022). FBL is currently hypothesized to serve as a central node in the network of immune responses to pathogens, and evidence suggests that this role may be evolutionarily conserved.

Notably, a correlation has been observed between small nucleoli and decreased FBL expression levels with longevity across various species (Proshkina et al., 2020). FBL may regulate longevity and the innate immune response through the modulation of nucleolar size equilibrium, although further comprehensive experiments are needed to substantiate this hypothesis. Such investigations hold promise for the development of novel approaches to enhance cellular innate defense mechanisms and promote overall health.

## 7 FBL and cancer

Cancer cells frequently exhibit nucleolar size imbalances and quantity abnormalities, potentially as a result of increased proliferation and metabolic demands. Given its involvement in ribosomal pre-rRNA processing, FBL has emerged as a promising therapeutic target for certain cancers, including breast cancer (El Hassouni et al., 2019). Extensive evidence indicates that FBL, a crucial player in ribosome biogenesis, can impact diverse cellular regulatory mechanisms, pathological progression, and aging processes. FBL exerts a dual influence on cancer development through its impact on ribosomal structure and mRNA translation efficiency, as well as its interaction with cancer-related genes to modulate cell cycle progression (Barneche et al., 2000; Semashko et al., 2012; Luo et al., 2022).

Ribosomal biosynthesis plays a role in cell growth and proliferation, and contributes to tumorigenesis (El Hassouni et al., 2019). Research has found that FBL has the potential as a therapeutic target, thereby reducing the genotoxic effects of anticancer therapy (Marcel et al., 2013; Shubina et al., 2018; El Hassouni et al., 2019; Barros-Silva et al., 2021; Sun et al., 2023). Both *in vivo* and *in vitro* experiments have shown that reducing the expression of FBL to non-tumor cells can significantly inhibit tumor growth (Decle-Carrasco et al., 2021).

It has been proved that in breast cancer and prostate cancer, FBL can promote cancer cell proliferation by regulating mRNA translation and controlling rRNA methylation (El Hassouni et al., 2019; Zhang et al., 2021). Overexpression of FBL can lead to changes in rRNA methylation patterns, impaired translation fidelity, and increased internal ribosome entry sites (IRES) of key cancer genes (Yi et al., 2021). Hassouni emphasized the correlation between FBL and cancer ribosomal biogenesis, and proposed that targeting FBL can reduce the toxic effects of anticancer therapy, making it a promising cancer treatment strategy (El Hassouni et al., 2019).

FBL can affect cell cycle progression by interacting with different cancer related genes. Research has found that p53 can inhibit its expression by directly binding to FBL. Although the effect of p53 on ribosomal synthesis is not yet clear, ribosomal biogenesis is overactivated in p53 activated cancer cells. Therefore, it is speculated that p53 may serve as a guarantee for protein synthesis by regulating FBL and subsequent ribosome generation and intrinsic activity (Su et al., 2014). Ribosome biogenesis is overactivated in cancer cells with p53 inactivation (Marcel et al., 2013). p53 inhibits FBL expression by binding directly to FBL. On the other hand, p53 activation by FBL knockdown is regulated not only by the ribosomal protein-MDM2-mediated protein stabilization pathway but also by increased PTB dependent, cap-independent translation (Su et al., 2014). In endometrial carcinoma, upregulated SNORD60 binds FBL, catalyzes the 2′-O-Me modification of PIK3CA mRNA and modulates the PI3K/AKT/mTOR signaling pathway to promote the development of endometrial cancer (Wu et al., 2023). Further research has found that the carcinogenic effects can be reversed by knocking down FBL or PIK3CA.

**FIGURE 7:**
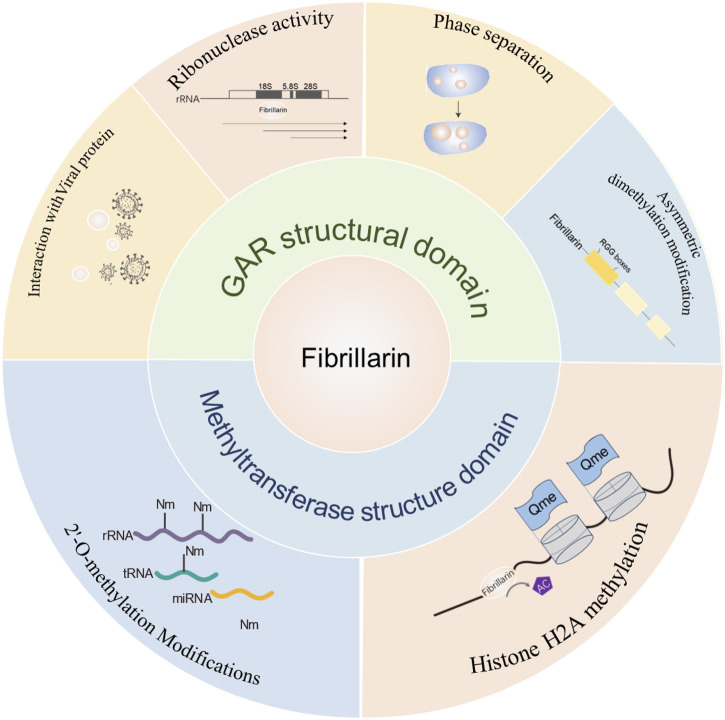
Summary diagram of FBL domain and function. The main functions of the GAR structural domain of FBL include: interactions with viruses, ribonuclease activity, phase separation and arginine dimethylation; the main functions of the methylase activity structural domain include: 2-O methylation of rRNA and methylation of histone H2A.

The structure‒function analysis of FBL showed that both major structural domains of FBL are capable of functioning in the cell (Figure 7), and we further generalized its role in cellular homeostasis, reproduction, inflammation and autoimmunity, and provided a detailed description of the pattern of FBL-mediated chemical modification of rRNAs (partly through p53-mediated modulation of the ribosome biosynthetic machinery) in cancer cells, opening up the possibility of developing anticancer molecules that target these “cancer ribosomes”.

## 8 Conclusion

The nucleolar protein Fibrillarin has important biological functions in rRNA modification, maintaining nucleolar homeostasis, reproductive development, and driving nucleolar phase transition. The function of FBL depends on its conserved methyltransferase domain and diversified N-terminal GAR domain. FBL can mediate the immune response process triggered by pathogen invasion. This review outlines the current body of knowledge of FBL and its involvement in other nucleolar components, LLPS events and their direct relationship with biological processes.
